# Production of Highly Active Extracellular Amylase and Cellulase From *Bacillus subtilis* ZIM3 and a Recombinant Strain With a Potential Application in Tobacco Fermentation

**DOI:** 10.3389/fmicb.2020.01539

**Published:** 2020-07-21

**Authors:** Jingcheng Dai, Aijun Dong, Guoxi Xiong, Yaqi Liu, Md. Shahdat Hossain, Shuangyuan Liu, Na Gao, Shuyang Li, Jing Wang, Dongru Qiu

**Affiliations:** ^1^Institute of Hydrobiology, Chinese Academy of Sciences, Wuhan, China; ^2^Technology Research Center of China Tobacco Hubei Industry Co., Ltd., Wuhan, China; ^3^College of Fisheries and Life Science, Dalian Ocean University, Dalian, China; ^4^University of Chinese Academy of Sciences, Beijing, China; ^5^National Institute of Biotechnology, Dhaka, Bangladesh

**Keywords:** amylase, cellulase, heterogeneous expression, bacillus, tobacco

## Abstract

In this study, a series of bacteria capable of degrading starch and cellulose were isolated from the aging flue-cured tobacco leaves. Remarkably, there was a thermophilic bacterium, *Bacillus subtilis* ZIM3, that can simultaneously degrade both starch and cellulose at a wide range of temperature and pH values. Genome sequencing, comparative genomics analyses, and enzymatic activity assays showed that the ZIM3 strain expressed a variety of highly active plant biomass-degrading enzymes, such as the amylase AmyE1 and cellulase CelE1. The *in vitro* and PhoA-fusion assays indicated that these enzymes degrading complex plant biomass into fermentable sugars were secreted into ambient environment to function. Besides, the amylase and cellulase activities were further increased by three- to five-folds by using overexpression. Furthermore, a fermentation strategy was developed and the biodegradation efficiency of the starch and cellulose in the tobacco leaves were improved by 30–48%. These results reveal that *B. subtilis* ZIM3 and the recombinant strain exhibited high amylase and cellulase activities for efficient biodegradation of starch and cellulose in tobacco and could potentially be applied for industrial tobacco fermentation.

## Introduction

Tobacco (*Nicotiana tabacum* L.) is the most economically important non-food cultivated product worldwide. China is the largest producer and consumer of tobacco in the world, accounting for about one third of the total global consumption each year (Liu et al., 2015). The unaged tobacco leaves are inadequate for cigarette products because of their sharp odors and undesirable aromas as well as harsh, irritating smoke (Huang et al., 2010). A further process called fermentation or aging for about 1–2 years is purposefully used to improve tobacco qualities. Fermentation not only shortens the aging cycle but also prominently develops the aroma and other qualities desirable in cigarette products (Su et al., 2011). This process not only is a chemical reaction process but also is linked to the enzymatic actions of microbes (Reid et al., 1937), which play extremely vital roles in the aging process. For example, many microbes, such as the genera *Aspergillus*, *Bacillus*, *Staphylococci*, *Penicillium*, and *Mucor*, initially have been observed upon cured and fermenting tobacco (Reid et al., 1937). Many previous researches have demonstrated the importance of microbial fermentation to the tobacco leaves for cigarette-making purposes (Di Giacomo et al., 2007; Zhao et al., 2007; Gong et al., 2009). In recent years, the high-throughput sequencing technologies have been utilized to analyze the diversity and dynamics of microbial communities from different types of tobaccos based on the 16S rRNA gene sequences (Huang et al., 2010; Su et al., 2011; Wang et al., 2018). However, little is known about the microorganisms appropriate for assisting in tobacco aging and microbial roles in aging, which are required for controlling artificial fermentation of tobacco.

Starch and cellulose are essential components of tobacco leaves, which have around 50% carbohydrates (10–30% starch, 10–25% cellulose, and 12% pectin) and 5–15% proteins, which affect the quality of flue-cured tobaccos (Reid et al., 1937). In a burning cigarette, tobacco leaf components are exposed to the burning environment; meanwhile, cellulose could lead to the emission of harsh smoke, resulting in a bitter taste among smokers. Some studies involved in the analysis of pyrolysis products from a single-component cellulose present in the tobacco leaves showed that some kinds of small molecular aldehyde and polycyclic aromatic hydrocarbons (PAHs) had been identified in tobacco smoke (Yang et al., 2018). PAHs are thought to have toxic properties such as carcinogenicity and cytotoxicity. The starch could affect the combustion velocity and completeness of cut tobacco and can also interfere with aroma-forming reactions because of the undesirable charring smell generated when the starch burned. Similarly, proteins could also produce throat choking and an unpleasant smell when burned (Reid et al., 1937). Therefore, the appropriate degradation of starch, cellulose, and proteins is the key to improve the quality of tobacco leaves. Meanwhile, the Maillard reaction is a chemical reaction between amino acids (the degradation products of proteins) and reducing sugars (the degradation products of starch and cellulose) that could produce distinctive flavor compounds and high-temperature speeds up the Maillard reaction when cigarettes are burning (Arihara et al., 2017).

In this study, a highly amylolytic and cellulolytic strain was directly isolated from the aging flue-cured tobacco and was found to be capable of simultaneously degrading starch and cellulose at a wide range of temperature and pH values. Starch and cellulose degradation were further confirmed by genome sequencing and comparative genomics analyses. The molecular genetics analyses and enzymatic activity assays demonstrate that both amylase and cellulase were secreted into ambient environment to function and could be heterologously overexpressed to improve the biodegradation of starch and cellulose in strain SIM1. The further small-scale fermentation experiment showed that the recombinant strain could improve the starch and cellulose degradation efficiency in tobacco, paving the way for tobacco fermentation application.

## Materials and Methods

### Tobacco Samples, Culture Conditions, and Plasmids

A total of three kinds of the aging flue-cured tobaccos were obtained from Sichuan, Yunnan, and Zimbabwe, respectively. The LB medium (5 g/L yeast extract, 10 g/L tryptone, and 10 g/L NaCl, pH 7.0), R2A agar medium (10 g/L tryptone, 5 g/L beef extract, 5 g/L NaCl, pH 7.0, containing 2% starch or 3% skimmed milk) for identification of amylase and proteinase activities, and modified minimal salt media (MSM) [0.5 g/L KCl, 0.5 g/L (NH_4_)_2_SO_4_, 1 g/L KH_2_PO_4_, 3.5 g/L Na_2_HPO_4_,2H_2_O, 0.2 g/L MgCl_2_,6H_2_O, 0.05 g/L Ca(NO_3_)_2_,4H_2_O, 0.001g/L FeSO_4_, 7H_2_O, and 1 ml of trace element solution] were used for isolation and cultivation of starch, cellulose, and protein-hydrolyzing bacteria. The *Escherichia coli–Bacillus* sp. shuttle vector, pMK3 (ampicillin resistance in *E. coli* and kanamycin resistance in *B. subtilis*), was used to construct the amylase and cellulose producing plasmid. The empty vector and resultant constructs were transferred into the *Bacillus* strains via electroporation. Plasmids used in this study are listed in Table 1.

**TABLE 1:** Plasmids used in this study.

**Plasmids**	**Description**	**Source or reference**
pUCP20T	Shuttle vector Amp^r^	H.P. Schweizer
pUCP20T-phoA(SP)	The *E. coli phoA* gene cloned in pUCP20T	This study
pUCP20T-phoA(NSP)	The modified *phoA* gene [phoA(NSP)], without the 5′-sequence encoding the N-terminal signal peptide, cloned in pUCP20T	This study
pUCP20T-amyE1(SP)-phoA(NSP)	The *amyE1*-*phoA* fusion gene, with the 5′-sequence encoding the N-terminal signal peptide fused with *phoA(NSP)*, cloned in pUCP20T	This study
pUCP20T-celE1(SP)-phoA(NSP)	The *celE1*-*phoA* fusion gene, with the 5′-sequence encoding the N-terminal signal peptide fused with *phoA(NSP)*, cloned in pUCP20T	This study
pUCP20T-xlnC(SP)-phoA(NSP)	The *xlnC*-*phoA* fusion gene, with the 5′-sequence encoding the N-terminal signal peptide fused with *phoA(NSP)*, cloned in pUCP20T	This study
pUCP20T-xlnA(SP)-phoA(NSP)	The *xlnA-phoA* fusion gene, with the 5′-sequence encoding the N-terminal signal peptide fused with *phoA(NSP)*, cloned in pUCP20T	This study
pUCP20T-licA(SP)-phoA(NSP)	The *licA*-*phoA* fusion gene, with the 5′-sequence encoding the N-terminal signal peptide fused with *phoA(NSP)*, cloned in pUCP20T	This study
pET28a	Expression vector with T7lac promoter	Novogene
pET28a-amyE1	Overexpression construct of *amyE1*	This study
pET28a-celE1	Overexpression construct of *celE1*	This study
pMK3	*E. coli–Bacillus* shuttle plasmid; ampicillin resistance in *E. coli* and kanamycin resistance in *B. subtilis*	
pMK3-amyE1	The *amyE1* gene cloned in pMK3	This study
pMK3-celE1	The *celE1* gene cloned in pMK3	This study

### Isolation of Starch and Cellulose Degrading Strains From the Aging Flue-Cured Tobaccos

After clipping the aging flue-cured tobacco, 5 g of each cut tobacco leaves was weighed and inoculated into 250-ml flasks containing 80 ml of MSM medium with 2.5% carboxymethyl cellulose (CMC) and 2% starch as a sole carbon source, respectively, and incubated with 220 rpm shaking at 28°C for 24 h. Subsequently, the bacterial culture was serially gradient diluted (10^–2^–10^–6^) and was plated on 2.5% CMC and 2% starch agars, incubated for 2–3 days at 30°C. Bacterial colonies with rapid growth and large diameters were picked and purified.

### Microbial Growth Assessment in Different pH and Temperature

The bacterial growth was measured by using the liquid LB medium. Four milliliters of liquid LB medium was prepared in one suit of three culture tubes (12 ml), and inoculated with 1% of the fresh bacterial inoculum (OD_600_ of 0.8) after autoclaving at 121°C for 20 min. The strains were cultured at a wide range of temperature (30–70°C) or pH (5.0–9.0) with 220 rpm shaking at suitable pH (7.0) or temperature (30°C) for 36 h. The bacterial growth was monitored by OD_600_ measurement.

### Determination of Enzymatic Activities

The chosen colony of individual strain was inoculated on 2.5% CMC and 2% of starch agar plates and incubated at 30°C for 16 h, respectively. For amylase activity tests, the plates were flooded with Lugol solution (gram iodine solution: 1% potassium iodide and 0.1% Iodine) for 5 min and positive activity was characterized by the clear zone formed on a purple background (Pascon et al., 2011). The cellulase and proteinase activity was assayed by measuring the CMC hydrolyzing zone on the CMC agar plates containing 0.2% Congo reds and the proteolysis zone on the skim milk agar plates after incubation for 36 h.

The amylase, cellulase, and proteinase activities were identified by using the culture supernatants of each strain over an entire growth period (36 h) using starch, CMC, and casein as substrates according to the methods of Miller (Miller, 1959; Miller et al., 1960) and McDonald and Chen (McDonald and Chen, 1965). The optical density of amylase and cellulase solutions was measured at 540 nm, respectively. The optical density of proteinase solutions was measured at 680 nm using a spectrophotometer. The enzymatic activities were measured by using Cellulase (CL) Assay Kit, α-amylase Assay Kit, and Alkaline Proteinase Assay Kit (Beijing Solarbio Science & Technology Co., Ltd). One unit (U) of amylase or cellulase activity was quantified as 1 μmol of glucose per minute generated by 1 ml of bacterial culture-produced enzymes under the assay conditions. One unit (U) of proteinase activity was quantified as 1 μmol of tyrosine per minute generated by 1 ml of bacterial culture-produced enzymes under the assay conditions. The enzymatic activity assays were carried out in triplicate.

### Effect of Temperature and pH on Amylase and Cellulase Activities and Stability

To evaluate the effects of pH on the enzymatic activity and stability, each bacterial culture was incubated at different pH values of 5.0–9.0 using the following buffer reaction solution: Tris–HCl buffer (pH 6.0–8.0), citrate buffer (pH 3.0–5.0), and glycine-NaOH buffer (pH 9.0–10.0); 2.5% starch and 2.0% CMC were used as substrates, respectively. The temperature should be kept at 30°C. The pH value of the cellulase and amylase reaction mixture was adjusted by using the above specified buffers, while both enzymatic activities were quantified as per the standard assay method. The stability of both the enzymes was determined by pre-incubating the enzyme reaction mixture for 30 min at 30°C. Meanwhile, the effects of temperature on the enzymatic activity and stability were determined by incubating each reaction solution at a different temperature (30–70°C) and the pH value should be kept at 7.0 for 1 day and the relative enzymatic activities were measured by the use of 2.5% starch and 2.0% CMC as substrates. The thermal stability of both the enzymes were quantified by pre-incubating the reaction mixtures at 30–70°C temperature and constant pH value of 7.0 for 30 min. The amount of enzyme synthesized was then quantified to analyze the thermal stability of both of the specified enzymes (Awasthi et al., 2018).

### Identification of Isolated Bacterial Strains

The genomic DNA of each isolated strain was extracted by using the E.Z.N.A.^®^ Bacterial DNA Kit (Omega Bio-Tek, United States). DNA was suspended in 100 μl of sterile distilled water and the DNA quality and quantity were checked by agarose gel electrophoresis and nano-drop measurement. The extracted DNA was used as template for PCR amplification of 16S rDNA by universal primers set: forward 27F primer (5′-AGAGT TTGATCCTGGCTCAG-3′) and reverse 1492R primer (5′-GG TTACCTTGTTACGACTT-3′) (Moreno et al., 2002). The sequence determined in this study was compared with the 16S rDNA sequences of the GenBank database. Nucleotide sequences were aligned initially using Clustal X (Thompson et al., 1997) and then adjusted manually. Distance matrices and phylogenetic trees were calculated according to the Kimura two-parameter model (Kimura, 1980) and the neighbor-joining algorithm (Saitou and Nei, 1987) using the MEGA 7 software packages (Kumar et al., 2016). One thousand bootstraps were performed to assign confidence levels to the nodes in the trees.

### Genome Sequencing, Assembly, Annotation, and Comparative Genomic Analyses

The DNA sequencing and assembly of the strain *B. subtilis* ZIM3 genomes were generated by using the Illumina Hiseq 2000 platform and SPAdes Genome Assembler^1^ (An et al., 2016). The open reading frames (ORFs) and the functional annotation of translated ORFs were predicted by using the RAST (Rapid Annotation using Subsystems Technology) server online^2^ (Overbeek et al., 2014).

The progressive Mauve (Darling et al., 2010) was used to compare the newly sequenced genome to the previously reported *Bacillus tequilensis* KCTC 13622, as a tool to check for synteny and unique regions among large blocks of genomic sequences. We compared the metabolic reconstruction of *B. subtilis* ZIM3 to that of *B. tequilensis* KCTC 13622 by using a function-based comparison tool (Brettin et al., 2015). Easyfig was used to visualize the coding regions and reveal the inter-cluster relationship (Sullivan et al., 2011).

### Alkaline Phosphatase A-Fusion Assay

The 5′-nucleotide sequence, encoding the signal peptide, of *amyE1*, *celE1*, *xlnC*, *xlnA*, and *licA* gene, was fused with the truncated *phoA* gene without the sequence encoding the N-terminal signal peptide, respectively, to determine the cellular protein location. Primers used in this study are listed in Supplementary Table S1. The alkaline phosphatase A (PhoA)-fusion assay was conducted according to the previous protocol (Hoffman and Wright, 1985; Dai et al., 2015).

### Expression, Purification, and Activity Assays of Amylase and Cellulase

The amylase genes *amyE1* (lacking the N-terminal sequence encoding the signal peptide, LNSP) and *celE1* (LNSP) were cloned into the pET28a vector and then were transferred into *E. coli* DE3 cells. The transformants of DE3 were incubated in the liquid LB medium (100 μg/L of ampicillin) at 37°C to an OD_600_ value of about 0.6–0.8 and were transferred into an incubator supplemented with IPTG (0.01%, w/v) at 16°C overnight to induce gene expression. The harvested cells were decomposed and homogenized by sonication (Scientz-II D, Ningbo). The recombinant proteins were purified by using Ni-NTA columns according to the previous reported protocol (Dai et al., 2019). The activities of amylase and cellulase were assayed by using Cellulase (CL) Assay Kit and α-amylase Assay Kit (Beijing Solarbio Science & Technology Co., Ltd). One unit (U) of enzyme activity was quantified as the amount of enzyme that synthesized against 1 μmol of glucose per minute under the assay conditions. The enzyme concentrations were measured by using a total protein assay kit (Jiancheng Biotech, Nanjing, China). Primers used in this study are listed in Supplementary Table S1.

### Cellulase and Starch Content Analysis

The flue-cured tobaccos were treated with 2 ml of distilled water, the strain ZIM3 culture, and the recombinant strain ZIM1 culture, respectively, for 6–8 days at 40°C temperature and 65% relative humidity. After that, the tobacco tissues were dealt with 70% ethanol and 100% acetone, and then air-dried at 37°C. Each 6- to 10-mg sample was incubated in 1 ml of Updegraff reagent (acetic acid:nitric acid:H_2_O, 8:1:2) for 30 min in boiling water. After washing twice with deionized water, the pellet was soaked in 1–2 ml of 67% H_2_SO_4_ for 1 h. The amount of glucose was measured by the colorimetric method at 620 nm using 0.3% anthrone as a dye. After that, the cellulose content was calculated according to the standard curve of D-glucose (Updegraff, 1969). For the determination of starch content, the treated tobaccos were dehydrated in a refrigerated vacuum evaporator operated at an air pressure of 8.1 kPa at −60°C for 24 h. Starch was extracted by percolation with 40% perchloric acid, followed by ultrasonic extraction for 10 min. The starch content was estimated by the continuous flow method (Song et al., 2016).

### Statistical Analysis

All experiments were conducted in three replicates. The means and standard deviation (SD) values are shown in the figures. One-way ANOVA was employed to determine statistical differences among multiple groups and *t*-test was applied to determine that between two groups.

## Results and Discussion

### Isolation and Identification of Potential Bacteria Producing Amylase and Cellulase

**FIGURE 1:**
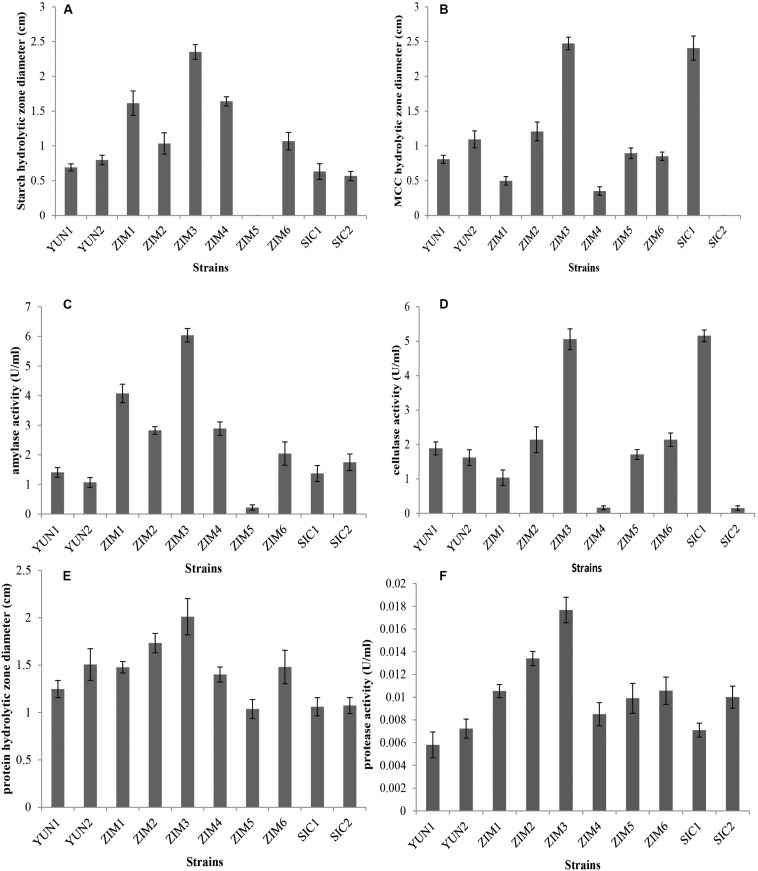
The diameter of starch **(A)** and CMC **(B)** hydrolytic zone and the amylase **(C)** and cellulase **(D)** activities of all selected isolates cultivated in modified MSM medium with 2.5% CMC and 2% starch as the sole carbon source and cultured at 28°C for 24 h. One unit (U) of enzyme activity was quantified as 1 ml of bacterial liquid produced enzyme that synthesized against 1 μmol of glucose per minute under the assay conditions. The diameter of protein hydrolytic zone **(E)** and the proteinase **(F)** activity of all selected isolates cultivated in medium for identification of proteinase activity and incubated at 28°C for 24 h. Error bars represent standard deviation.

There were 39 strains obtained from Yunnan tobacco, Sichuan tobacco, and Zimbabwe tobacco leaves through selective culture. Among these isolates, 10 strains resulted in the hydrolytic zone of different sizes on the MSM agar medium containing 2.5% CMC and 2% starch. The cellulose and starch degradation efficiency of two strains (YUN1 and YUN2) isolated from tobacco leaves from Yunnan province, two strains (SIC1 and SIC2) from Sichuan province, and six strains (ZIM1, ZIM2, ZIM3, ZIM4, ZIM5, and ZIM6) from Zimbabwe was evaluated based on the hydrolytic zone diameter (cm). The strain SIC1 exhibited a larger hydrolytic zone diameter on CMC agar plate, and the strains ZIM1 and ZIM4 produced wider hydrolytic zone diameters on starch agar plate (Supplementary Figure S1). Interestingly, the strain ZIM3 simultaneously resulted in a larger hydrolytic zone diameter on both CMC and starch agar plate (Figures 1A,B). Meanwhile, in our *in vivo* assays, the activity of amylase and cellulase was measured after 24 h of bacterial incubation during which the extracellular hydrolyzing enzymes were produced and secreted (Figures 1C,D). However, there were no significant differences in the protease activity observed among these strains (Figures 1E,F). These results indicated that the measured amylase activity, 2846 U/g, and cellulase activity, 2448 U/g, of the strain ZIM3 were more than 2.5 times as high as those of other strains except for the strain SIC1 whose cellulase activity was about 2560 U/g. These results are in accordance with the findings of English et al. (1967) who isolated eight strains of *B. subtilis*, five strains of *B. coagulans*, four strains of *B. megaterium*, and three strains of *B. circulans* from fermented Connecticut broadleaf tobacco. Some of those strains were employed to enrich the normal thermophile flora of “sweating” tobacco (English et al., 1967). However, there was no further research on the strains producing amylase and cellulose. Later, Awasthi et al. (2018) observed that four potential amylase- and cellulose-producing bacteria were isolated from the food wastes compost and belonged to the amylolytic and cellulolytic strains. Gonzalez Pereyra et al. (2020) also found that aflatoxin-degrading *Bacillus* strains could degrade zearalenone, which also produced proteases, amylases, and cellulases.

Interestingly, some strains could not form colonies on CMC agar plate but exhibited the hydrolytic zone diameter after incubation for 2–3 days. We suspected that they might enter into a viable but non-culturable (VBNC) state. Some studies have shown that VBNC bacteria still possessed metabolic activity such as nitrogen removal capabilities in indigenous population of the sediments (Su et al., 2019). Similarly, the PAH-degrading *Novosphingobium* sp. LH128 enters a VBNC-like state upon inoculation into soil but is metabolically active (Fida et al., 2017).

### Effects of Temperature and pH on Growth of Bacterial Strains

**FIGURE 2:**
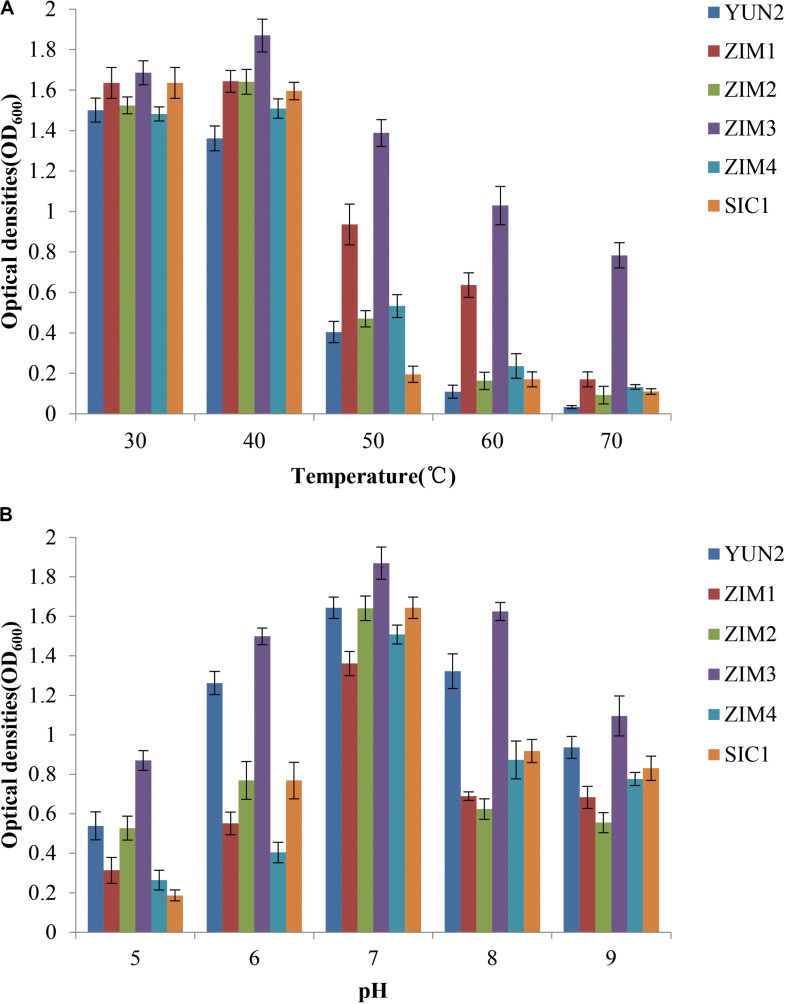
Growth of bacterial strains under different temperature and pH values. Effects of **(A)** temperature (30–70°C) and **(B)** pH (5.0–9.0) on the growth of the six strains. These strains were incubated in LB culture medium with shaking (220 rpm) for 36 h. Error bars represent standard deviation.

To characterize the effects of temperature and pH values on bacterial growth, a total of six cellulolytic and amylolytic strains were selected according to their higher enzymatic activities. Bacterial growth profile of different strains was evaluated after 36 h under the different temperature or pH value. The results showed that the strains ZIM1 and ZIM3 exhibited significantly higher cell density than other strains at 40–70°C and pH 5.0–9.0 (Figures 2A,B). Besides, the previous results also showed the strain ZIM3 had higher amylase and cellulase activities. Together, these results confirmed that the strain ZIM3 did have a potential ability to biodegrade CMC and starch into the sub-products like glucose under the conditions of 5.0–9.0 pH and 40–70°C temperature. The strain ZIM3 possesses highly thermostable starch and cellulose hydrolytic capacity under different pH values and temperatures. Our results are consistent with the findings of Awasthi et al. (2018), who isolated some thermotolerant strains from the food waste compost, such as *Brevibacillus borstelensis* and *Bacillus thuringiensis*, which achieved significantly higher cell density at the high temperature of 50–60°C and pH 6.0–8.0. Thus, we propose that the strain ZIM3 would be considered as a biological agent to improve the efficiency of tobacco fermentation based on its better adaptability at a wide range of temperature and pH values.

### The Phylogenetic Analysis of the Strain *B. subtilis* ZIM3

**FIGURE 3:**
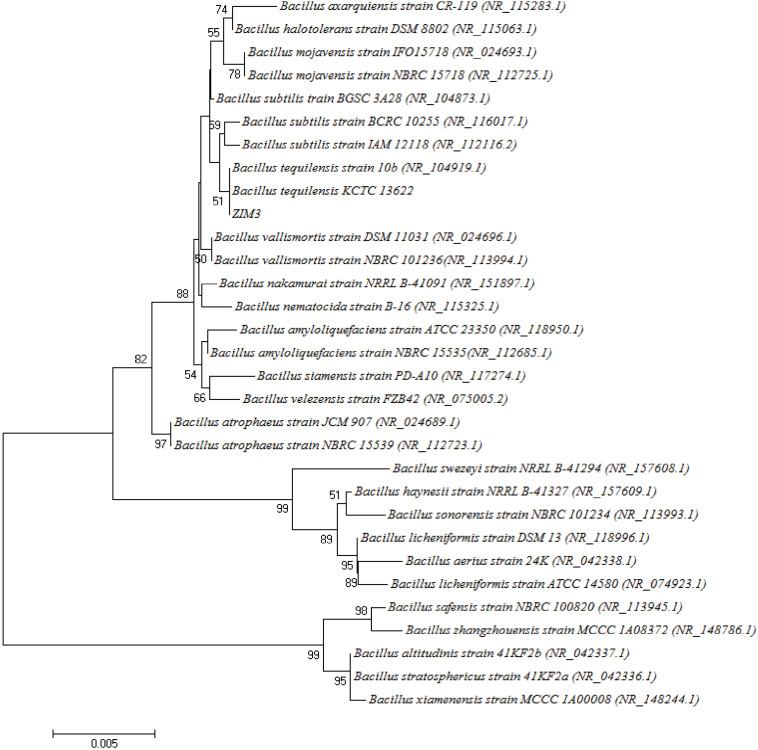
Neighbor-joining tree of the strain ZIM3 and related bacterial strains based on the neighbor-joining algorithm of the 16S rDNA sequences. Bootstrap values are shown as percentages of 1000 replicates. The bar (0.005) at the bottom of the tree indicates the substitution per nucleotide position.

Based on the 16S rDNA sequence analysis, the strains ZIM1 (*Bacillus amyloliquefaciens*, 98%), ZIM2 (*Bacillus zhangzhouensis*, 99% identity), ZIM3 (*Bacillus subtilis*, 98%), ZIM4 (*B. amyloliquefaciens*, 99%), ZIM5 (*Bacillus* sp., 99%), ZIM6 (*B. zhangzhouensis*, 99%), SIC1 (*Bacillus stratosphericus*, 98%), SIC2 (*Paenibacillus amylolyticus*, 98%), YUN1 (*Solibacillus silvestris*, 98%), and YUN2 (*Bacillus kochii*, 98%) had been identified and were found to be closely related to the previously classified *Bacillaceae* species, respectively. Because of the high amylase and cellulase activity, a phylogenetic tree of the strain ZIM3 based on 16S rRNA gene sequences is constructed (Figure 3) and it was found that the strain ZIM3 was closely related to the previously described *B. tequilensis* KCTC 13622 and *B. tequilensis* 10b strains. *B. tequilensis* possessed a novel extracellular active thermo-alkali-stable laccase, an alkaline pectate lyase, and a solvent-stable amylase, with potential applications in biobleaching, xenobiotics bioremediation, food industry, decolorization of textile dyes, and plastic degradation (Chiliveri and Linga, 2014; Tiwari et al., 2014; Sondhi et al., 2015). However, to date, few researches focus on the highly thermostable cellulase and amylase activity of *Bacillaceae* species isolated from the flue-cured tobaccos.

### Comparative Genomic Analyses of the Strain *B. subtilis* ZIM3 and *B. tequilensis* KCTC 13622

The genome sequencing and annotation of the strain *B. subtilis* ZIM3 were carried out to reveal the mechanism underlying its higher amylase and cellulase activity. The sequence data were generated in the sequencing of the genomic DNA library of the strain ZIM3 and the genome was assembled *de novo* from 14,316,557 paired end reads, with the length of 150 nucleotides (nt) and approximately 500^∗^ coverage. The genome of the strain ZIM3 was estimated to be 4073 kilo-base pairs (bp) in length, with a G + C content of 43.7% and 4195 predicated coding sequences, while the genome size of the *B. tequilensis* KCTC 13622 strains was estimated to be 3981 kilo-bases, with a 43.9% GC content and 4363 predicated coding sequences.

**FIGURE 4:**
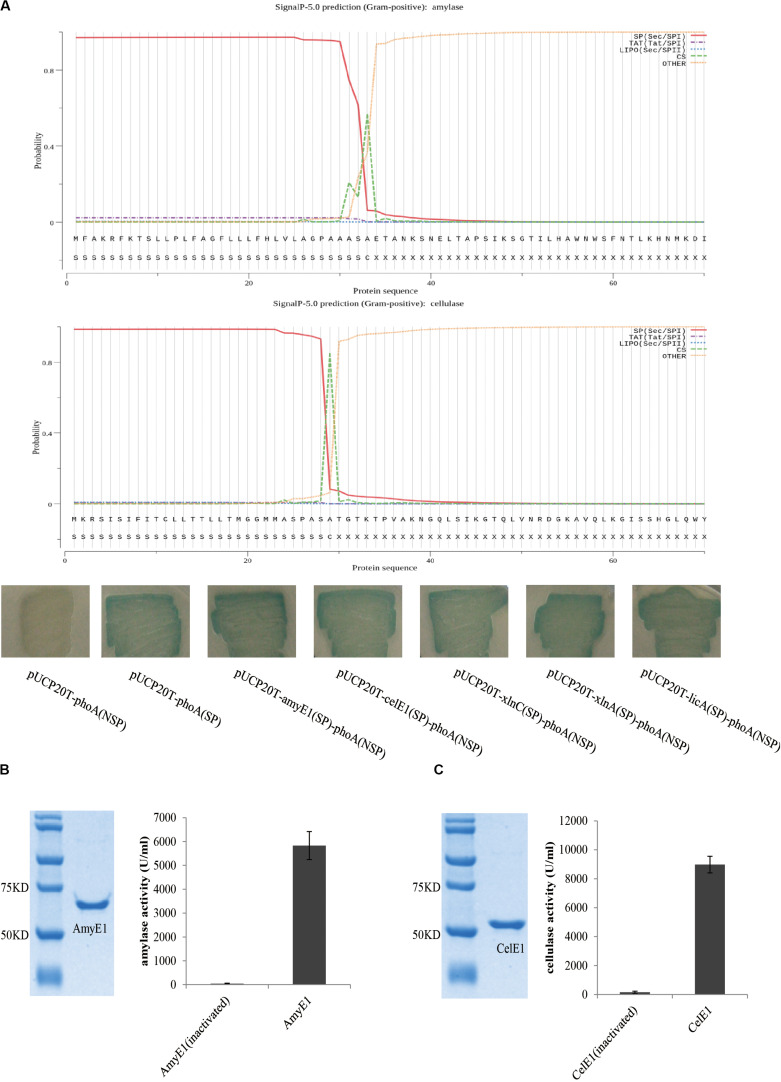
Amylase and cellulase activity assays of AmyE1 and CelE1. **(A)** PhoA-fusion assay (the lower panel) demonstrated that AmyE1 and CelE1 are secreted into the periplasm as computationally predicted (two upper panels). The two upper panels are computational prediction of signal peptide for AmyE1 and CelE1 secretion by using the SignalP 5.0. The 5′-nucleotide sequence, encoding the signal peptide of *amyE1*, *celE1*, *xlnC*, *xlnA*, and *licA* gene, was fused with the *E. coil phoA* gene without the sequence encoding the N-terminal signal peptide, respectively. **(B)** Photographic representation of the SDS-PAGE of AmyE1 proteins. Lane 1: marker proteins; lane 2: enzyme supernatant (amylase). Molecular weights were presented in the form of kDa. The amylase activity was defined under the assay conditions described. **(C)** Photographic representation of the SDS-PAGE of CelE1 proteins. Lane 1: marker proteins; lane 2: enzyme supernatant (cellulase). Molecular weights were presented in the form of kDa. The cellulase activity was defined under the assay conditions described.

The comparative genomics analysis of the two strains *B. subtilis* ZIM3 and *B. tequilensis* KCTC 13622 was conducted. Multiple genome alignments of these two genomes, using progressive Mauve (Supplementary Figure S2), demonstrated a few similarities between *B. subtilis* ZIM3 and *B. tequilensis* KCTC 13622. However, there were some rearrangements and sequence elements specific to a particular genome, respectively. By using a function-based comparison tool, we observed that both *B. subtilis* ZIM3 and *B. tequilensis* KCTC 13622 possessed a cellulolytic and hemi-cellulolytic multienzyme complex and an amylolytic multienzyme system, indicating that they could degrade complex plant biomass into fermentable sugars efficiently. We found that these two strains possess a variety of plant biomass-degrading enzymes, including alpha-amylase AmyE1 (EC 3.2.1.1), oligo-1,6-glucosidase GluA (EC 3.2.1.10), alpha-glucosidase GluB (EC 3.2.1.20), neopullulanase NplT (EC 3.2.1.135), beta-1,4-glucanase (cellulase) CelE1 (EC 3.2.1.4), glucuronoarabinoxylan endo-1,4-beta-xylanase XlnC (EC 3.2.1.136), endo-1,4-beta-xylanase XlnA (EC 3.2.1.8), endo-beta-1,3-1,4 glucanase (licheninase) LicA (EC 3.2.1.73), and spore coat protein CotA (Laccase) (EC 1.10.3.2). In particular, the alpha-amylase AmyE1, beta-1,4-glucanase (cellulase) CelE1, glucuronoarabinoxylan endo-1,4-beta-xylanase XlnC, endo-1,4-beta-xylanase XlnA, and the endo-beta-1,3-1,4 glucanase LicA were computationally predicted to contain the signal peptide for secretion by using the SignalP 5.0 software (Armenteros et al., 2019) and Protter (Omasits et al., 2014) (Figure 4A). Meanwhile, the PhoA-fusion assay demonstrated that AmyE1, CelE1, XlnC, XlnA, and LicA were secreted into the periplasm of *E. coli* DH5 alpha as predicted above, because the signal peptide of AmyE1, CelE1, XlnC, XlnA, and LicA could mediate the secretion of PhoA into periplasm of *E. coli*, respectively (Figure 4A and Supplementary Figure S4). Comparative genomic analyses revealed that *B. subtilis* ZIM3 possess more functional genes than *B. tequilensis* KCTC 13622, which lacks approximately 605 genes such as the genes required for chitin and *N*-acetylglucosamine utilization, histidine degradation, spore germination, teichoic and lipoteichoic acid biosynthesis, inositol catabolism, lactate utilization, as well as cold and heat shock response (Supplementary Table S2). Furthermore, the relatedness of these two strains was also determined, using the Easyfig software (Supplementary Figure S3). The alignment analysis demonstrated that the close relationship and the extensive gene rearrangements between *B. subtilis* ZIM3 and *B. tequilensis* KCTC 13622 might account for their speciation and adaptation to the specific habitats in which they evolved. The *B. subtilis* ZIM3 has been sent and stored in the China Center for Type Culture Collection (CCTCC NO: M2018707).

### The Genes Potentially Involved in Cellulose and Starch Degradation

To further characterize the enzyme activities of AmyE1 and CelE1, a series of expression, purification, and *in vitro* activity assays of the AmyE1 and CelE1 recombinant proteins were conducted. The alpha-amylase AmyE1 (residues 34–659, lacking the N-terminal signal peptide, MFAKRFKTSLLP LFAGFLLLFHLVLAGPAAASA) gene and beta-1,4-glucanase (cellulase) CelE1 (residues 30–499, lacking the N-terminal signal peptide, MKRSISIFITCLLTTLLTMGGMMASPASA) gene were successfully cloned into the pET28a vector and were overexpressed in the *E. coli* DE3 strain (Figures 4B,C). *In vitro* assays demonstrated that the recombinant AmyE1 protein exhibited an amylase activity as high as 5827 U/ml and the cellulase activity of overexpressed CelE1 reached 8978 U/ml (Figures 4B,C). These results, together with the *in vivo* assays (Figure 1), strongly indicated that both AmyE1 and CelE1 are functional in the strain *B. subtilis* ZIM3 and are probably secreted outside of the cell to degrade the cellulose and starch. Some psychrotolerant yeasts only exhibited extracellular amylase or cellulase activity at a narrow range of low temperature and pH values (Carrasco et al., 2016), unlike *B. subtilis* ZIM3, which could adapt to a wider range of temperature and pH values. Interestingly, there were previous attempts to employ the thermostable cellulase- and amylase-producing bacteria for the biodegradation of tobacco waste extract and food wastes (Awasthi et al., 2018; Ye et al., 2019). In this study, we attempted to improve the quality of tobacco leaves by biodegradation of starch, cellulose, and protein in flue-cured tobacco leaves via fermentation, and hopefully could produce distinctive flavor. Some other researches showed that *Bacillus* could produce extracellular xylanase (Khandeparker et al., 2017) and extracellular thermo-alkali-stable laccase (Sondhi et al., 2015), which are consistent with our genomic prediction on the strain *B. subtilis* ZIM3, with an exception for the laccase of strain ZIM3, which was intracellular bacterial spore coat protein. The 1,3-1,4-β-glucanase of *Bacillus* exhibited remarkable activities (Teng et al., 2006; Wang et al., 2014; Niu et al., 2017, 2018) that can effectively hydrolyze high-molecular-weight β-glucans into oligosaccharides by cutting the 1,4-β glycosidic bonds (Niu et al., 2017), promising for application in the fermentation industry.

### Effects of Temperature and pH on Enzymatic Activities in Liquid Culture

**FIGURE 5:**
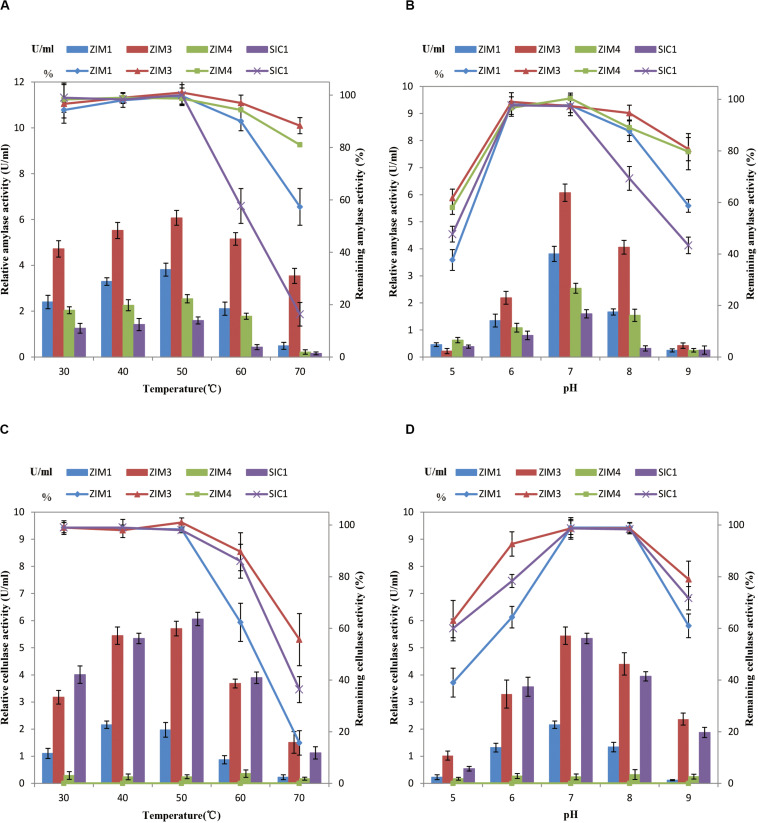
Effects of temperature **(A)** and pH **(B)** on amylase activity and stability after incubation for 30 min. Effects of temperature **(C)** and pH **(D)** on cellulase activity and stability after incubation for 30 min. One unit (U) of enzyme activity was quantified as 1 ml of bacterial liquid produced enzyme that synthesized against 1 μmol of glucose per minute under the assay conditions. Error bars represent standard deviation.

To further characterize the enzymatic activities and stability of the strain *B. subtilis* ZIM3 as compared to other strains at a wide range of temperature and pH values, a series of experiments were conducted to measure the cellulase and amylase activities over the temperature and pH range from 30 to 70°C, and 5.0 to 9.0, respectively. The four selected strains all showed high enzymatic activities at 37°C (Figure 1). However, our results indicated that the SIM3 strain not only had a highly active amylase activity but also maintained the maximum amylase stability over a wide range of temperature (30–70°C) and pH values (6.0–9.0) (Figures 5A,B). The maximum enzymatic activities were observed at 50°C and pH 7.0. These results are consistent with the previous finding that a strain of *B. amyloliquefaciens* produced the highest enzyme activity at about 50°C (Tanyildizi et al., 2007). Similarly, the strain of *Bacillus* sp. GM8901 exhibited highly active amylase stability at pH 6.0–8.0 (Hmidet et al., 2009). Moreover, the strains ZIM1 and ZIM4 have lower amylase activities but higher amylase stability (Figure 5). Besides, the strains SIM3 and SIC1 exhibited maximum cellulase activities and stability at a wide range of temperature and pH values (Figures 5C,D), and the maximum enzymatic activities were observed at the temperature of 50°C and pH 7.0 (Figures 5C,D), consistent with the findings of the higher cellulase activity of another *Bacillus* sp. strain observed at pH 7.0–8.0 (Mawadza et al., 2000). In *Bacillus pumilus*, the cellulase activity was retained about 70% for 24 h cultivation at 70°C (Poorna and Prema, 2007). Interestingly, the strain SIC1 has lower cell density as compared to other strains at 50–70°C. Therefore, the strain SIM3 could really grow quickly and produce the extracellular highly active cellulase and amylase at a wide range of temperature (30–70°C) and pH values (6.0–9.0).

### Improving Activities of Amylase and Cellulase by Genetic Engineering

**FIGURE 6:**
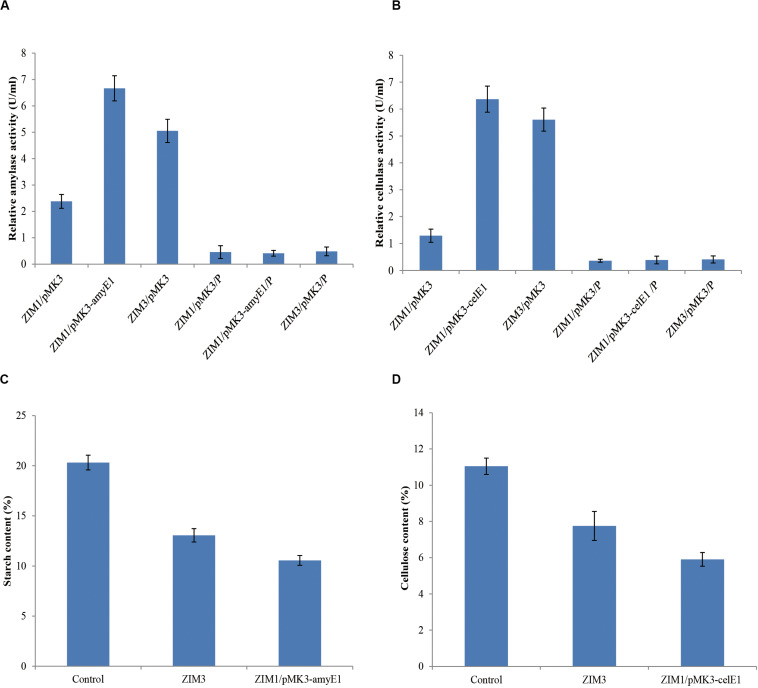
Overexpression of the *amyE1* **(A)** and *celE1* **(B)** in the strain ZIM1 could improve the amylase and cellulase activities and the biodegradation efficiency of starch **(C)** and cellulose **(D)** in tobacco. ZIM1/pMK3 represents the strain ZIM1 carrying the pMK3 plasmid, and the enzyme activities from cell-free culture supernatants were measured; note that ZIM1/pMK3/P represents the strain ZIM1 carrying the pMK3 plasmid, and the enzyme activities from the cell pellet extract (P) were measured; Control represents the treatment with 2 ml of distilled water; ZIM3 represents the treatment with 2 ml of ZIM3 strains cultures; ZIM1/pMK3-amyE1 represents the treatment with 2 ml of ZIM1/pMK3-amyE1 strains cultures; ZIM1/pMK3-celE1 represents the treatment with 2 ml of ZIM1/pMK3-celE1 strains cultures.

The strain ZIM1 achieved significantly higher cell density but lower amylase and cellulase activities as compared with other strains at higher temperature (Figure 2A). Therefore, we tried to further improve its efficiency for biodegradation of starch and cellulose by using the molecular genetics approaches. The results showed that overexpression of *amyE1* (from the strain ZIM3) and *celE1* (also from the stain ZIM3) could lead to the increase of amylase and cellulase activities in strain ZIM1, respectively (Figures 6A,B). In particular, amylase activity was increased by threefold in the engineered strain carrying the pMK3-amyE1 compared to that of the wild-type strain ZIM1 carrying only empty pMK3 vector, even slightly more than that in strain ZIM3. Similarly, the cellulase activity was increased by five times in the recombinant strain carrying the pMK3-celE1 compared to the wild-type strain ZIM1. Moreover, the activities of cellulase and amylase in the cell pellet were also measured, but the enzymatic activities were below the limit of detection (Figures 6A,B). Therefore, the findings, together with the above-described results, strongly indicated that most of the amylase and cellulase were secreted into the outside of cells and very low or negligible amount was within the cell. Importantly, we confirmed that the amylase and cellulase of strain ZIM3 exhibited higher enzymatic activities and was heterologously expressed to improve the biodegradation of starch and cellulose in strain SIM1. A study showed that expression of enzymes such as β-1,4-glucosidase and β-1,4-endoglucanase in the ethanologenic *E. coli* was found as better biomass hydrolysis (Munjal et al., 2015). Nowadays, many studies are mainly focused on bacteria strain itself producing thermostable amylase and cellulase enzymes, and these results showed that many wild-type strains could biodegrade the food waste, tobacco waste, and wastepaper (Poorna and Prema, 2007; Awasthi et al., 2018; Ye et al., 2019). However, few researches are involved in genetic approaches for improvement of microbial enzyme activity applied in tobacco fermentation. We firstly improve activities of amylase and cellulase by genetic engineering, paving the way for the engineered strain to be applied in tobacco fermentation.

### Effects of Microbial Treatment on the Cellulose and Starch Contents in Tobacco

Cellulose and starch, primary components of the tobacco cell organization and skeleton, are about 11 and 20% in tobacco, respectively (Figures 6C,D) (Reid et al., 1937). High cellulose and starch contents, which could decrease the tobacco flavor and smoke quality of cigarettes, lead to intense irritation of the sensory organs and coughing during smoking (Zhu et al., 2015). To reduce the cellulose and starch contents in tobacco, we evaluated the efficacy of the ZIM3 strain and the engineered strain ZIM1 to degrade cellulose and starch in tobacco. As described above, the wild-type strain ZIM3 and the engineered strain ZIM1 have significantly higher amylase and cellulase activities, respectively (Figures 6A,B). In the present study, a small-scale tobacco fermentation experiment was also performed with our strains. The flue-cured tobaccos were further treated with the strain ZIM3 or (and) the engineered strain ZIM1 for 6–8 days at 40°C temperature and 65% relative humidity. As shown in Figures 6C,D, the contents of starch and cellulose in tobacco declined significantly after the treatment by the strain ZIM3 and the engineered strain ZIM1. Interestingly, the content of starch in tobacco treated with strain ZIM3 and ZIM1 was reduced by 35.7 and 48%, respectively. On the other hand, the content of cellulose in tobacco was reduced by 29.8 and 46.6%, respectively. In conclusion, treatment with the strain ZIM3 and the engineered strain ZIM1 could improve the starch and cellulose degradation efficiency by 30–48%. These findings are consistent with the fact that overexpression of *amyE1* and *celE1* could lead to the increase of amylase and cellulase activities in strain ZIM1, hereby improving the biodegradation efficiency of starch and cellulose in tobacco. It was previously shown that the tobacco stems treated with a pectinase solution led to an increase in the contents of reducing sugars and Maillard reaction products by 20.5 and 67.2%, respectively (Zhu et al., 2015). The neutral aroma substances were increased by 29.94%, which could improve the aroma quality of tobacco leaves and the overall sensory quality to a great extent (Zhu et al., 2015). These results showed that *B. subtilis* ZIM3 and the recombinant strain exhibited high amylase and cellulase activities and hopefully could be applied in tobacco fermentation for an efficient degradation of both starch and cellulose.

## Data Availability Statement

The original contributions presented in the study are publicly available. This data can be found in NCBI, accession number: PRJNA636601.

## Author Contributions

JD carried out the experiments and analyzed the experimental raw data. AD and GX designed the experiment. YL, SLiu, and NG executed the experiment process and recorded the data. SLi and JW contributed to sample collection. MH, JD, and DQ wrote and revised the manuscript. All the authors read and approved the final manuscript.

## Conflict of Interest

An application for the Chinese invention patent has been submitted and is under review. AD and GX were employed by Hubei Industry Co., Ltd.

The remaining authors declare that the research was conducted in the absence of any commercial or financial relationships that could be construed as a potential conflict of interest.

## Footnotes

1 http://cab.spbu.ru/software/spades/

2 https://rast.nmpdr.org/
