# Supplementary material for: Production of Highly Active Extracellular Amylase and Cellulase From Bacillus subtilis ZIM3 and a Recombinant Strain With a Potential Application in Tobacco Fermentation
Source: Front Microbiol. 2020 Jul 21;11:1539. doi: 10.3389/fmicb.2020.01539 (PMC7385192; doi:10.3389/fmicb.2020.01539)

**Supplemental Figure S1** CMC, starch and protein hydrolyzing zone of the all selected isolates cultivated on the modified minimal salt medium (MSM) agar plates, and incubated at 28 °C for 24-36hrs.


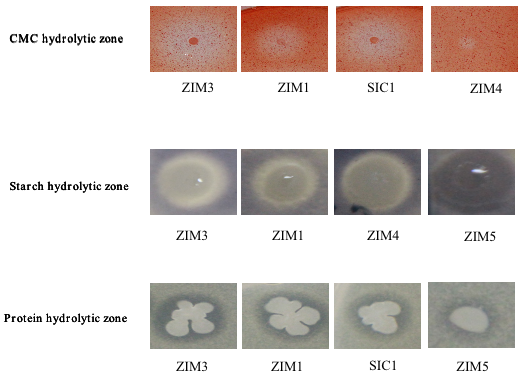


**Supplemental Figure S2** Mauve alignment of the annotated complete genomes of *Bacillus* sp. ZIM3 and *Bacillus tequilensis* KCTC 13622. The degree of sequence similarity between regions is given by a similarity plot within the colored blocks with the height of the plot proportional to the average nucleotide identity. Also, the regions with a lack of homology are outside these blocks or shown in white inside the blocks.


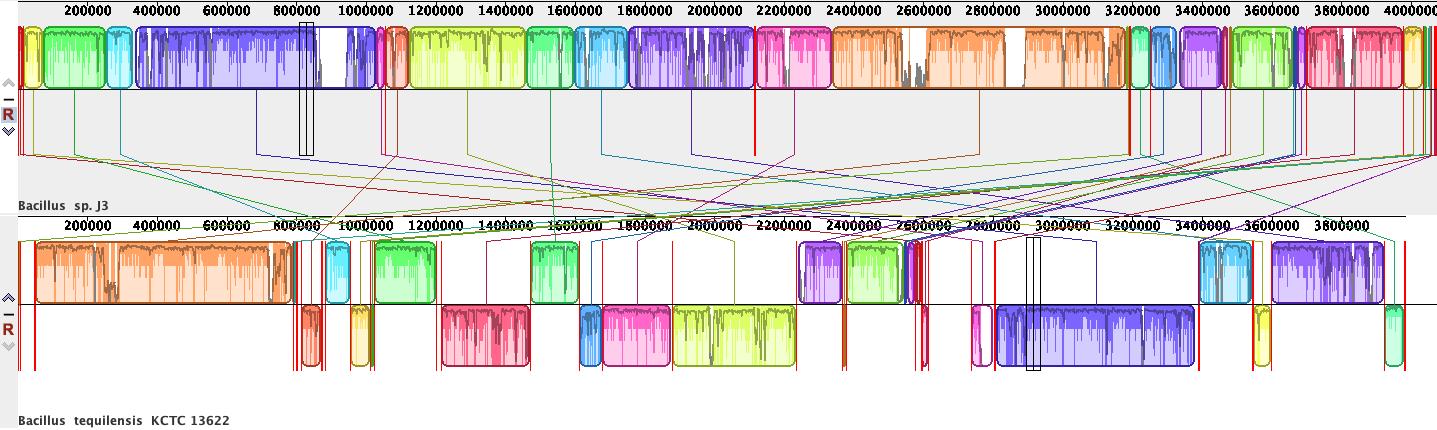


**Supplemental Figure S3** The comparison of the complete genome sequence of *Bacillus* sp. ZIM3 (top) and *Bacillus tequilensis* KCTC 13622 (bottom) using Easyfig. The colored arrows indicate ORFs according to their predicted function. The homologous regions between phages are indicated by gray shading.


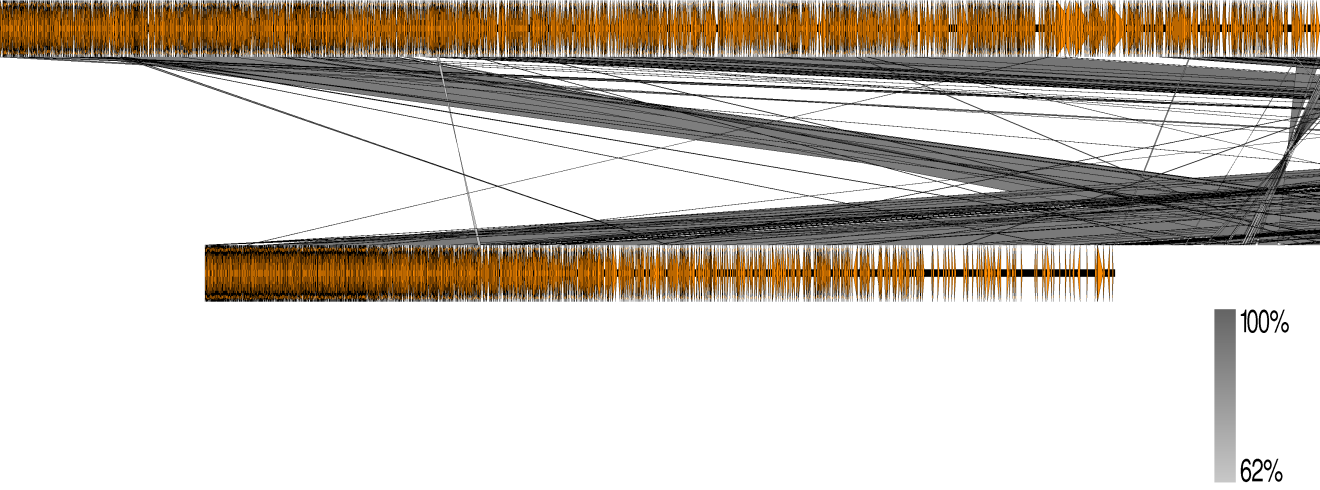


**Supplemental Figure S4** The alkaline phosphatase A (PhoA)-fusion assay demonstrated that the AmyE1, CelE1, XlnC, XlnA and LicA are secreted into the periplasm as computationally predicted (5 upper panels) because the fused signal peptide of AmyE1, CelE1, XlnC, XlnA and LicA did result in the positive activity of PhoA which must be located in the aerobic periplasm for proper conformation (2 lower panels) in the phoA null strain Escherichia coli DH5α, respectively. The upper panel is computational prediction of signal peptide for AmyE1, CelE1, XlnC, XlnA and LicA secretion by using the SignalP5.0 and Protter softwares, respectively.

**AmyE1 protein:**


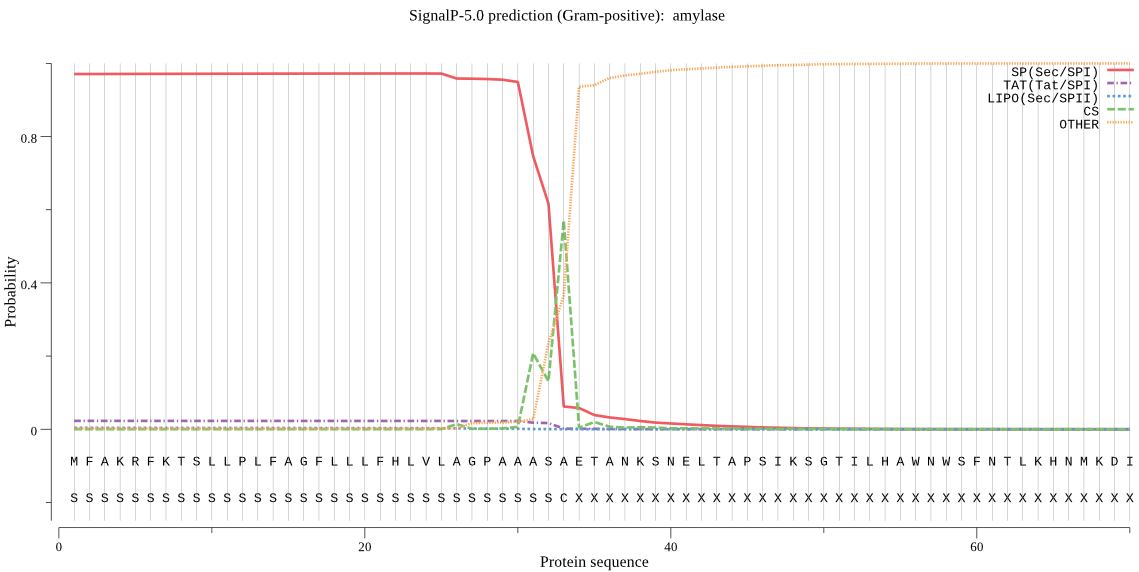


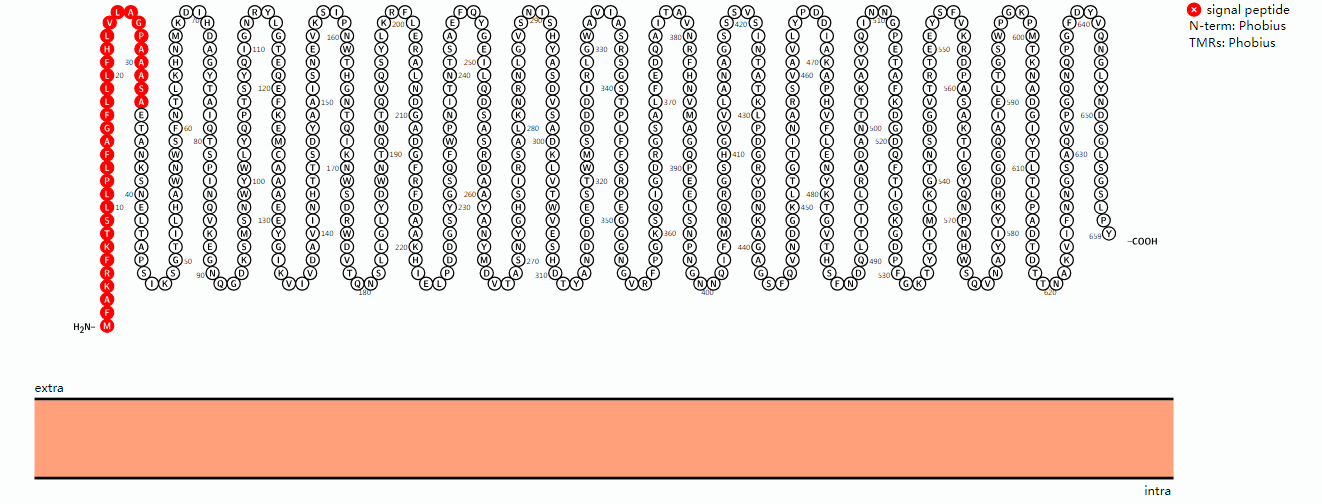


**CelE1 protein:**


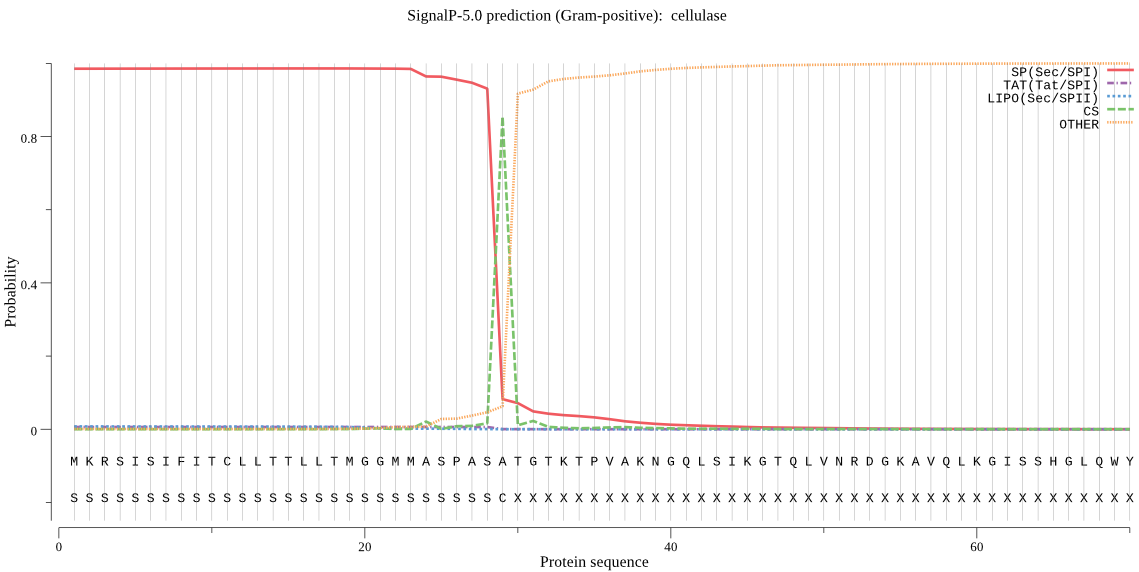


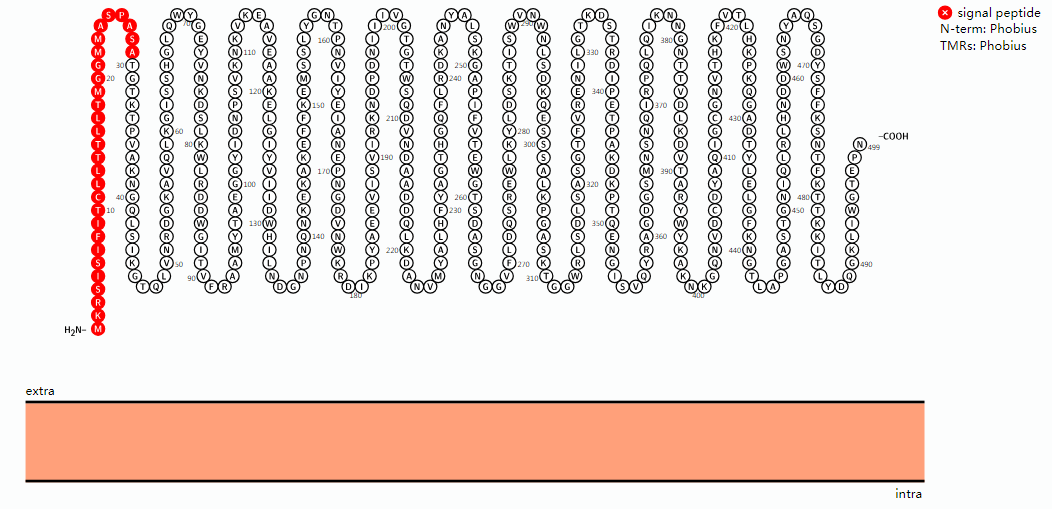


**XlnC protein:**


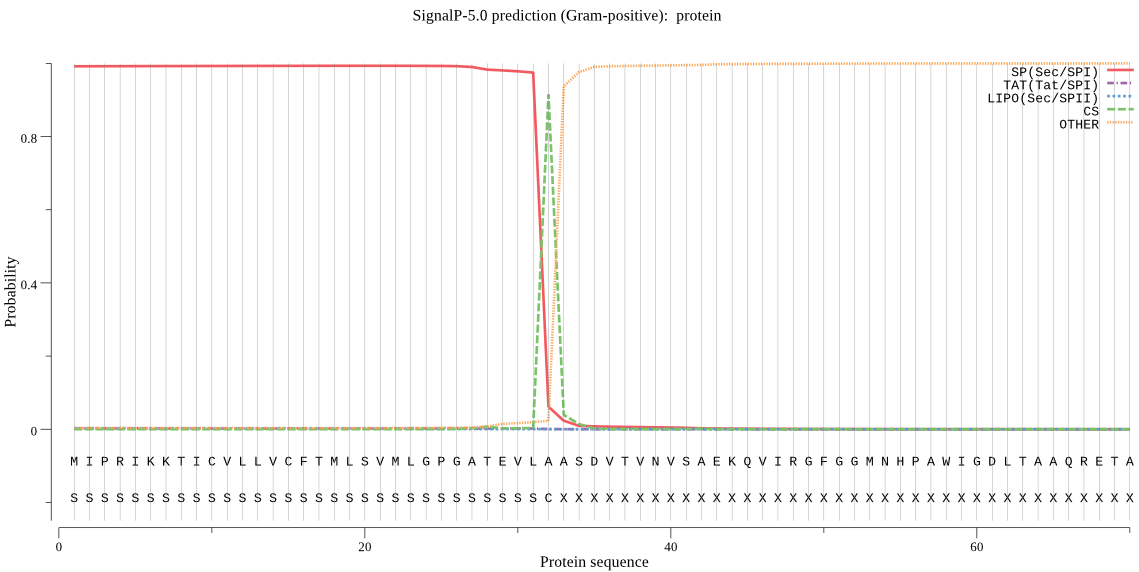


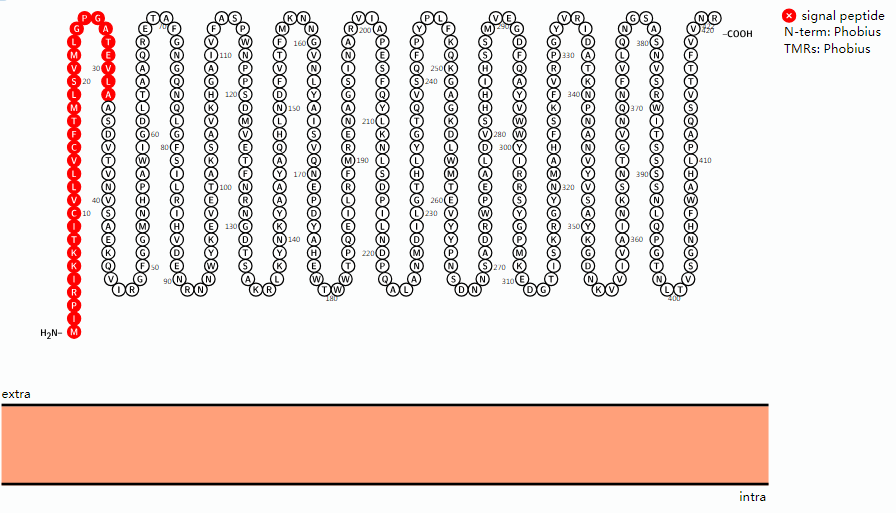


**XlnA protein:**


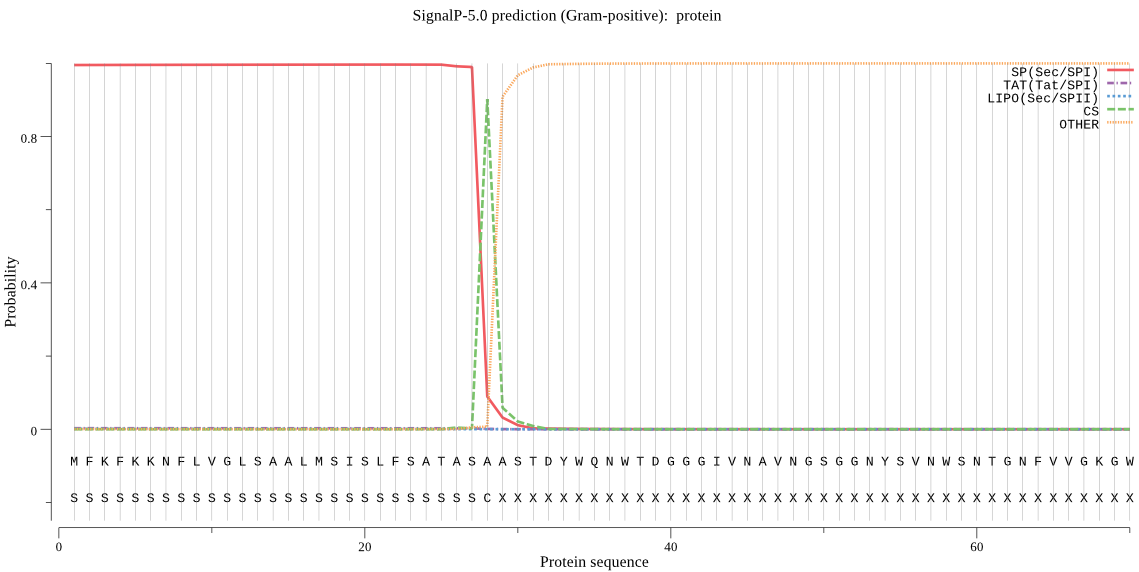


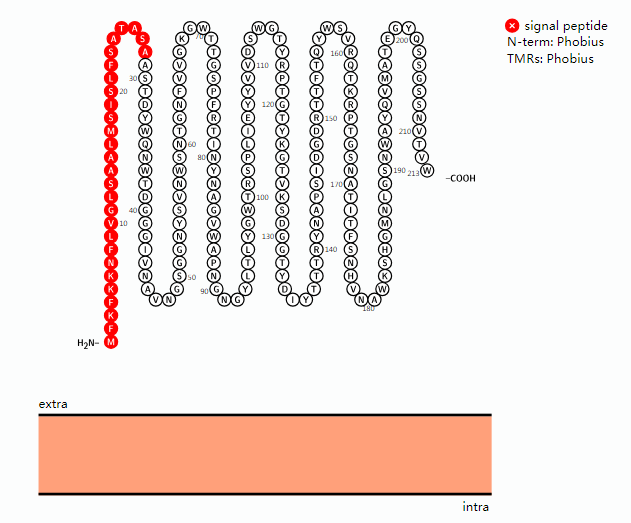


**LicA protein:**


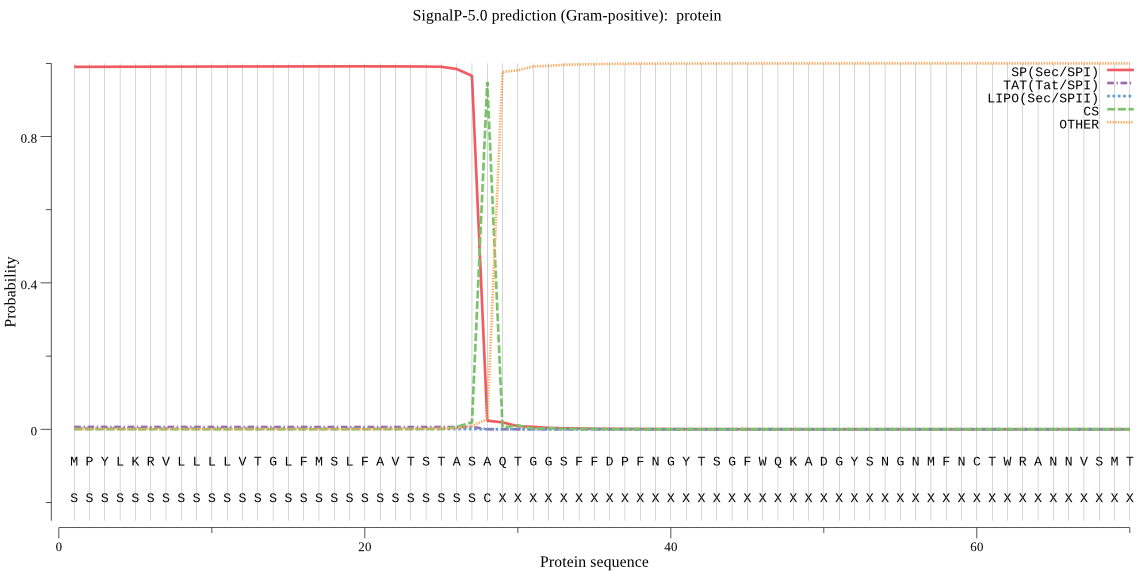


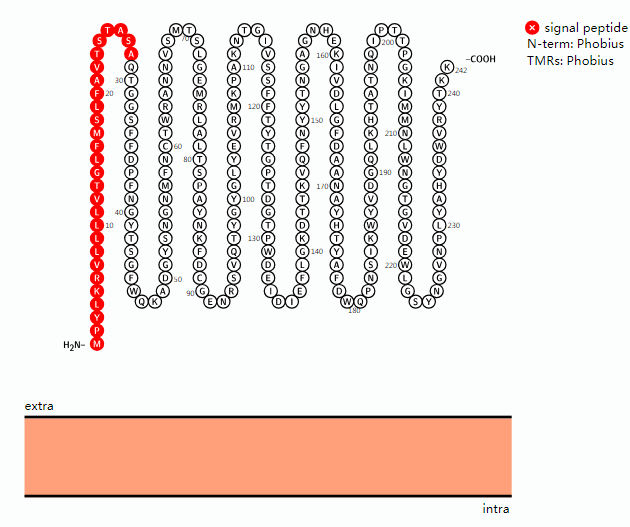


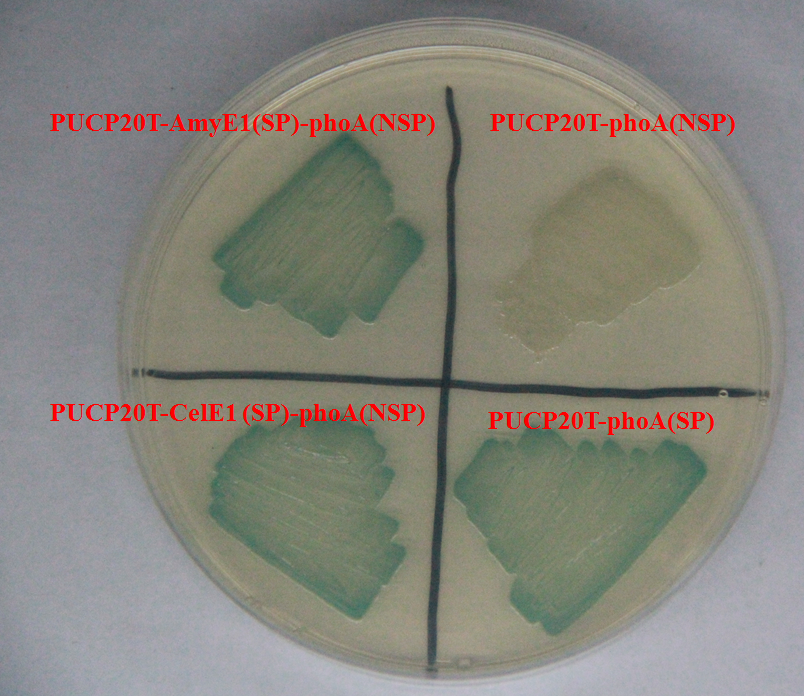


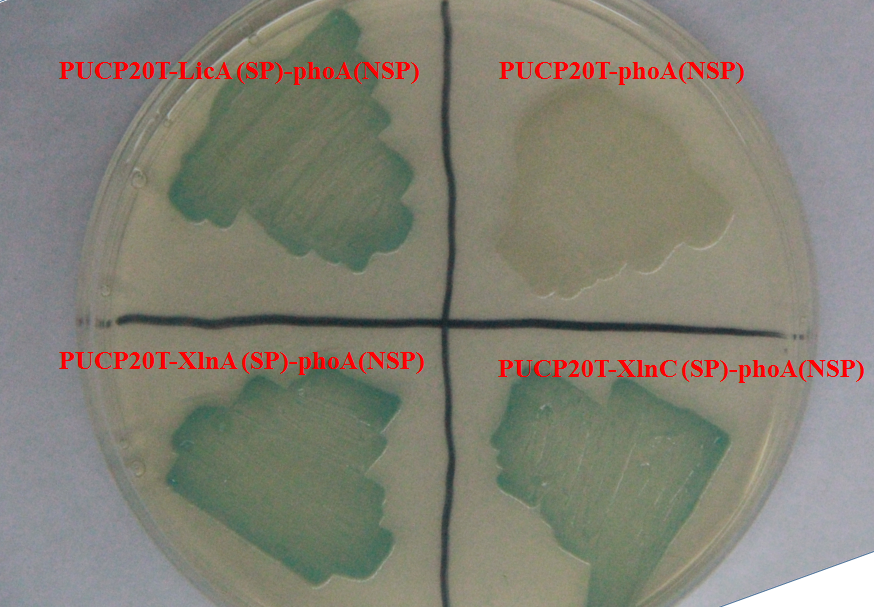

Supplement: Supplementary file 1 [file Data_Sheet_1.docx]
