# Supplementary material for: Production of Highly Active Extracellular Amylase and Cellulase From Bacillus subtilis ZIM3 and a Recombinant Strain With a Potential Application in Tobacco Fermentation
Source: Front Microbiol. 2020 Jul 21;11:1539. doi: 10.3389/fmicb.2020.01539 (PMC7385192; doi:10.3389/fmicb.2020.01539)
Supplement: Supplementary file 2 [file Data_Sheet_2.docx]

**Supplemental Table S1** Primers used in this study

| **Primer** | **Oligonucleotide sequence (5’-3’)** |
| --- | --- |
| amyE1 -F(SP) | 5’- CAGAGCTC GCGGAAGAATGAAGTAAGAG-3’ |
| amyE1-R(SP) | 5’- CAGGCATTTCTGGTGTCCGAGCACTCGCAGCCGCCGGTC-3’ |
| amyE1-phoA-F(NSP) | 5’- GACCGGCGGCTGCGAGTGCTcggacaccagaaatgcctg-3’ |
| celE1-F(SP) | 5'- CAGAGCTCagtgaagagccaaaatgatg-3' |
| celE1-R(SP) | 5'- CAGGCATTTCTGGTGTCCGTGCTGATGCCGGCGAAGCC-3' |
| celE1-phoA-F(NSP) | 5'- ggcttcgccg gcatcagcacggacaccagaaatgcctg-3' |
| xlnC-F(SP) | 5'- CAGAGCTC AGCCCGCCATTATGGATATG-3' |
| xlnC-R(SP) | 5'- CAGGCATTTCTGGTGTCCGTGCCAAAACTTCAGTAGCG-3' |
| xlnC-phoA-F(SP) | 5'- CGCTACTGAAGTTTTGGCAcggacaccagaaatgcctg-3' |
| xlnA-F(SP) | 5’- CAGAGCTCgtatacgggtgctgcctca-3’ |
| xlnA-R(SP) | 5’- CAGGCATTTCTGGTGTCCGTGCAGAGGCGGTTGCCGAAA-3’ |
| xlnA-phoA-F(SP) | 5’- tttcggcaaccgcctctgcacggacaccagaaatgcctg-3’ |
| licA-F(SP) | 5’- CAGAGCTC CTTTTAAAAGAATCATGTAA-3’ |
| licA-R(SP) | 5’- CAGGCATTTCTGGTGTCCGAGCCGAGGCAGTAGAAGTGAC-3’ |
| licA-phoA-F(SP) | 5’- GTCACTTCTACTGCCTCGGCTcggacaccagaaatgcctg-3’ |
| phoA-R(NSP) | 5’- GCTCTAGAGTTTTATTTCAGCCCCAGAG-3’ |
| PET28a-amyE1-F | 5’-tggtgccgcgcggcagccatatgGAAACGGCGAACAAATCGAA-3’ |
| PET28a-amyE1-R | 5’-tggtggtggtggtggtgctcgagtcaataaggaagagaaccgc-3’ |
| PET28a-celE1-F | 5’- tggtgccgcgcggcagccatatgacagggacaaaaacgccag-3’ |
| PET28a-celE1-R | 5’- tggtggtggtggtggtgctcgagCTAATTTGGTTCTGTTCCCC-3’ |
| pMK3-amyE1-F | 5’- CAAAGCTTGCGGAAGAATGAAGTAAGAG-3’ |
| pMK3-amyE1-R | 5’- CGGATCCtcaataaggaagagaaccgc-3' |
| pMK3-celE1-F | 5’- CAAAGCTTagtgaagagccaaaatgatg-3' |
| pMK3-celE1-R | 5’- CGGATCC GTTTTATTTCAGCCCCAGAG-3' |
| 27F | 5’-AGAGTTTGATCCTGGCTCAG-3’ |
| 1492R | 5’-GGTTACCTTGTTACGACTT-3’ |
